# Articles

**Published:** 1850-07

**Authors:** G. Y. Shirley


### ARTICLE VI.

Jacksonville, Ill., *January* 1850.

Messrs. Editors:

*Gentlemen:*—Remote as I am in the far West from advantages derivable from association, still I have an abiding interest in the progress of dental science, and wish the Journal continued. The standard of dentistry will never be sufficiently elevated in the West, nor its advantages fully known and realized, until a good operator is to be found in every country town. I know not how it is that while the cities are well supplied, very many important points in small towns are entirely destitute. And I know not how it is that so few are found to engage in the dental profession, while the candidates for the practice of medicine are found in excess, unless it be that the practice of the former does not admit of imposition so easily as the latter.

Dentists here, who deserve the name, are few and far between, and when of the right stamp in soul as well as professional skill, their visits are much like angels. There is a class who can only live by travelling. These are not only ignorant of the first principles of the profession, but what is worse, unwilling to learn, and are unprincipled. Some call them quacks and some tyros, but they seem more like a class who in the days of yore were called tinkers. The latter is now (I believe) extinct, and the former can only become so by showing the people what the dental profession now is.

Physicians are generally as ignorant of dentistry here, as the people they serve. One-half do not know enough to direct the people to the dentist when demanded. Having been engaged in medical practice for sixteen years, I am the better able to know the truth of what I say. There are those, however, who are not ignorant of the high stand dental surgery has taken, and fully appreciate its worth. While some seem to seek for more light on the subject, others refuse to come to it, lest their deeds be reproved. Two of our most respectable physicians kept a poor sufferer five years under medical treatment for what one, (a professor in a medical school (called neuralgia, and the other, chronic rheumatism. Their patient called on me to extract a tooth, after which he gave me a history of his case. As he was an intelligent man, I was very soon able to convince him he had chronic disease in the left antrum, and showed him Harris on the Maxillary Sinus. He immediately submitted to my treatment, and in one month was perfectly cured. Many other cases might be named, but I speak only of this one to prove what I asserted. This poor man was vesicated, mercurialized and narcotized by one who had snuffed his nose at having heard four gentlemen were employed in the Baltimore School of Dental Surgery, to teach what! he arrogantly enquired.

I consider dentistry one of the most useful of all professions, and when properly pursued, is blessed in what it takes, and what it gives. Hère, in the wide and prosperous west, is a very suitable place for the young practitioner to test this truth. I have patients who come from twenty to one hundred and twenty miles, and if there was a competent dentist in every village, in place of detracting from business it would make the more. In a pecuniary point of view it is a much better business than the practice of medicine. The increasing demand for it was the first reason to induce me to engage in it. I have thus given a few thoughts hastily on the subject of the profession, prompted by the interest I take in the cause, and not to bring myself into notice as a practitioner.

Very respectfully,

G. Y. Shirley.

